# The structural basis of chicken, swine and bovine CD8αα dimers provides insight into the co-evolution with MHC I in endotherm species

**DOI:** 10.1038/srep24788

**Published:** 2016-04-28

**Authors:** Yanjie Liu, Xin Li, Jianxun Qi, Nianzhi Zhang, Chun Xia

**Affiliations:** 1 Department of Microbiology and Immunology, College of Veterinary Medicine, China Agricultural University, Beijing 100094, China; 2 Key Laboratory for Insect-Pollinator Biology of the Ministry of Agriculture, Institute of Apiculture, Chinese Academy of Agricultural Sciences, Beijing 100093, China; 3 CAS Key Laboratory of Pathogenic Microbiology and Immunology, Institute of Microbiology, Chinese Academy of Sciences, Beijing 100101, China; 4 The Key Laboratory Zoonosis of Ministry of Agriculture of China, Beijing 100094, China

## Abstract

It is unclear how the pivotal molecules of the adaptive immune system (AIS) maintain their inherent characteristics and relationships with their co-receptors over the course of co-evolution. CD8α, a fundamental but simple AIS component with only one immunoglobulin variable (IgV) domain, is a good example with which to explore this question because it can fold correctly to form homodimers (CD8αα) and interact with peptide-MHC I (p/MHC I) with low sequence identities between different species. Hereby, we resolved the crystal structures of chicken, swine and bovine CD8αα. They are typical homodimers consisting of two symmetric IgV domains with distinct species specificities. The CD8αα structures indicated that a few highly conserved residues are important in CD8 dimerization and in interacting with p/MHC I. The dimerization of CD8αα mainly depends on the pivotal residues on the dimer interface; in particular, four aromatic residues provide many intermolecular forces and contact areas. Three residues on the surface of CD8α connecting cavities that formed most of the hydrogen bonds with p/MHC I were also completely conserved. Our data propose that a few key conserved residues are able to ensure the CD8α own structural characteristics despite the great sequence variation that occurs during evolution in endotherms.

The adaptive immune system (AIS) is a sophisticated defence network that recognizes and clears non-self antigens. The emergence of the AIS is symbolized by the appearance of its core molecules, such as the major histocompatibility complex class I (MHC I), T-cell receptors (TCRs), and the co-receptor CD8^1^,^2^,^3^,^4^. Over the course of evolution, these core components have exhibited large changes in their amino acid (AA) sequences and further enhanced the complexity of the AIS. However, how they maintain the initial structural and inherent characteristics and relationships with the receptors through long-term co-evolution remains unclear.

CD8 is expressed on the T-cell surface as dimers in two isoforms, the CD8αα homodimer and the CD8αβ heterodimer; both consist of an extracellular immunoglobulin variable (IgV) domain, a stalk region, a transmembrane domain and a cytoplasmic tail^5^,^6^. The CD8α and CD8β genes are related and closely linked within a locus of 36 Kb in mice and 56 Kb in humans, and their transcription and expression are regulated by numerous factors^7^. Although CD8αα and CD8αβ have similar binding affinities with peptide-MHC I (p/MHC I) and are equally recruited to the immunological synapse, they are expressed on different immune cells and play different key roles in cellular immunity. CD8αβ, expressed by αβ T cells, binds to p/MHC I by its extracellular domain and facilities Lck to phosphorylate the TCR-CD3 complex by its cytoplasmic tail, which can greatly enhance the sensitivity of specific cytotoxic T-cell (CTL) proliferation^8^,^9^,^10^,^11^. CD8αβ is believed to be the main co-receptor for T-cell activation and differentiation because it can enhance TCR sensitivity by approximately 100-fold over that of cells expressing only CD8αα^12^,^13^. The mechanisms are still unclear, but are postulated to relate to the shorter β stalk as well as glycosylation modifications and glycan adducts of CD8αβ^14^,^15^. The differences between CD8αα and CD8αβ in the stimulation of T cells maybe also relate to cholesterol- and glycosphingolipid-enriched membrane microdomains (lipid rafts). Lck and CD8αβ were mainly present in lipid rafts, whereas CD8αα was excluded^12^. The cytoplasmic portion of CD8β was found to mediate partitioning of CD8 in lipid rafts, where it efficiently associates with p56^lck^, and promotes raft association of TCR/CD3^16^. CD8αα is broadly distributed on γδT cells, NK cells, subsets of dendritic cells and intestinal intraepithelial lymphocytes^17^. The function of CD8αα is still enigmatic, and recent studies have suggested that CD8αα might be a negative regulator of T-cell activation^18^. In addition, in mouse, CD8αα can bind to the nonclassical MHC I molecule (TL) with greater affinity than to classical MHC I molecules^19^,^20^, and this interaction plays an important role in the differentiation of memory T-cell and mucosal T-cell immune responses^21^,^22^.

Although CD8αα and CD8αβ have great functional distinctions, crystallography studies show they are similar in structure and in the manner of binding p/MHC I. Unlike functional studies, the structural studies of CD8αα are clearer than those of CD8αβ. The first crystal structure of human CD8αα homodimers via association of its extracellular typical immunoglobulin variable domains was first resolved in 1992^23^. The subsequent crystal structures of mouse and monkey CD8α confirmed CD8αα homodimers similar among other mammals^24^,^25^. The mouse CD8αβ structure was resolved and showed a remarkable resemblance to CD8αα in size, shape and surface electrostatic potential^5^. To date, three complexes of CD8-p/MHC I with two different human MHC I alleles (HLA-A2 and A24) and one mouse MHC I (H-2K^b^) have been determined^5^,^26^,^27^. Analysis of these complexes revealed that although there are some species-specific recognition features, in both species, CD8αα homodimers contact MHC I α1-α3 domains and *β*2-microglobulin (*β*2m) and predominately bind to the protruding MHC I α3 domain CD loop in an antibody-like manner. The structures of mouse H-2D^d^ and CD8αβ were resolved recently^17^; the CD8αβ does not contact the MHC I α1, α2 domains and β2m, unlike the CD8αα, but it mainly binds the MHC I α3 domain CD loop in a manner similar to CD8αα. In addition, the crystal structures of TL and CD8αα revealed their strong affinity is strengthened through subtle structure changes in the TL α3 domain by the substitution of three contact residues^20^.

To date, the CD8 and MHC I genes have been found in a wide variety of species^1^. In addition to studies of human and mouse CD8 and MHC I, studies of the CD8 and MHC I of poultry and livestock, such as chicken, swine and bovine, have shown great progress in recent years. The cDNA sequences of chicken, swine and bovine CD8 have been cloned^28^,^29^,^30^,^31^. In addition, the two isoforms of the chicken CD8 dimer have been found to be the key markers to divide the subsets of T cells^3^. These CD8 molecules have their own unique species-specific characteristics. For example, chicken CD8α, but not CD8β, were polymorphic, and the majority of AA substitutions were located in the immunoglobulin V-like domain^30^. There was a unique subset of extra-thymic CD4^+^CD8^+^ double-positive (DP) cells in swine, which were believed to relate to the memory T-cell response, and CD8α chains were expressed abundantly on swine lymphocytes, mainly in the CD8αα homodimer isoform^32^,^33^. The majority of bovine milk lymphocytes were predominantly CD8^+^αβ-T cells and displayed the memory T-cell phenotype^34^. The crystal structures of chicken, swine and bovine p/MHC I molecules have been resolved recently^35^,^36^,^37^,^38^,^39^. These p/MHC I structures revealed that although the MHC I molecules from different animals have their own species-specific features, they have a similar overall architecture and structural basis to present peptides. However, no information about the crystal structures of chicken, swine and bovine CD8 molecules have been reported except the preliminary studies of these CD8 molecules by our group^40^,^41^,^42^.

During long-term co-evolution, CD8α AA sequences changed greatly in different species. It is still unclear how CD8αα molecules keep similar structures despite the extremely low sequence identities. In addition, the MHC I genes are polymorphic and show significant variation among different animals^43^. The maintenance of the molecular interaction between CD8αα and p/MHC I during long-term co-evolution has not been explained. Here, we report the high-resolution crystal structures of chicken, swine and bovine CD8α and confirm that they are capable of forming homodimers. The identities between chicken and mammalian CD8α were low; chicken CD8αα showed a unique helix and a short CDR2 loop as well as minimal inter-chain hydrogen bonding and dimer interface area. Furthermore, highly conserved residues were identified, and they were critical in the CD8αα structures. The dimerization of CD8αα mainly depended on the conserved residues on the dimer interface; in particular, four aromatic residues provided great intermolecular forces and contact areas. The residues of CD8αα and p/MHC I that formed most of the hydrogen bonds between them are highly conserved. Our results suggest that although CD8α and MHC I sequences change drastically during co-evolution, a few conserved key residues ensure that the CD8α forms dimers and interacts with p/MHC I during at least the co-evolution of endotherm species.

## Results

### The canonical CD8αα homodimers with distinct species-specific features

**Figure 1:**
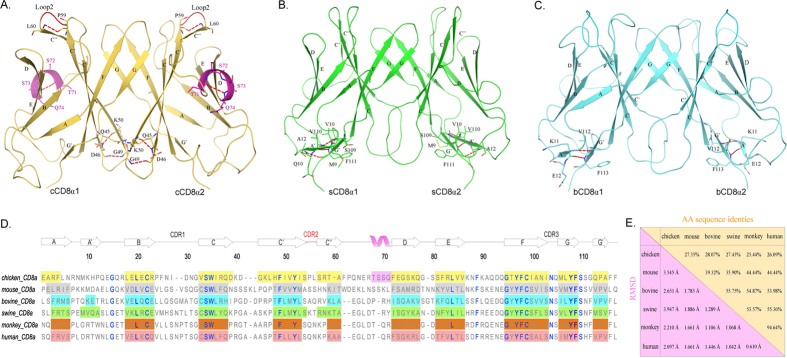
Structural characteristics and comparison of cCD8αα, sCD8αα and bCD8αα homodimers. The overall structures of cCD8αα, sCD8αα and bCD8αα and their distinct characteristics are shown in (**A**–**C**). The β-strands and specific residues are labelled. Hydrogen bonds are shown as red dashed lines. (**A**) The cCD8αα homodimer is coloured yellow-orange, and its unique helix is coloured light magenta; its extremely short CDR2-like loop, which only consists of 2 residues, is coloured red. (**B**) The sCD8αα homodimer is coloured green, and the composed residues of its additional A’ strand are displayed. (**C**) The bCD8αα homodimer is coloured cyan, and its A’ strand was also determined. (**D**) The AA alignment of CD8α molecules based on their crystal structures is shown. Each strand consisting of residues is labelled by a coloured box: yellow-orange for chicken, grey for mouse (PDB ID: 3DMM), green for swine, cyan for bovine, orange for monkey (PDB ID: 2Q3A) and salmon for human (PDB ID: 1CD8). The hollow arrow on the regions of boxes represents the corresponding strand, and the only helix in cCD8αα is also labelled by a light magenta box. The highly conserved residues are coloured blue. (**E**) The values of AA identities and RMSDs of the CD8α molecules whose structures have been resolved are shown.

Three extracellular Ig-like domains of chicken, swine and bovine CD8α (cCD8α, sCD8α and bCD8α) were crystallized and diffracted to 2.0 Å, 1.8 Å and 1.8 Å, respectively. All of them are CD8αα homodimers, and each CD8α exhibited a typical IgV architecture consisting of two anti-parallel sheets (Fig. 1A–C). The sheets of CD8α are composed of 10 β strands, but there are 11 β strands in sCD8α and bCD8α because their A and G strands are divided into two separate parts. The inner sheets of these CD8αα homodimers contain C, C’, C”, G and F strands, and the outer sheets consist of A, B, D and E strands. The detailed AA compositions of each strand in these different CD8α molecules are shown in Fig. 1D. Although the AA sequence identities of the resolved CD8α IgV domains are quite low (Fig. 1E), especially between the mammals and the chicken (non-mammal) (<30%), there are 17 conserved residues in all the CD8α molecules (blue). Among these residues, two C (in the B and F strands, respectively) form the critical disulphide bond of the Ig superfamily domains, and highly conserved residues - G (in the AB or A’B loop), L (in the B strand), Y (in the C strand), L (in the E strand), G and Y (in the F strand) – that compose the common core of the IgV domains can also be found in these CD8αα structures (Fig. 1D)^44^,^45^.

By comparing all the resolved CD8αα structures, the root-mean-square deviations (RMSDs) of the mammal CD8αα molecules are below 1.9 Å, which is lower than the RMSDs of the mammal CD8ααs and cCD8αα (Fig. 1E). Among these three structures, cCD8αα is a special one that exhibits unique characteristics. For example, cCD8αα has the longest C and C’ strands and a unique alpha helix between C” and D strands (Fig. 1A,D). These indicate there is an obvious gap between non-mammal and mammal CD8α molecules. The two artiodactyl CD8αα dimers also have some distinct structural characteristics, and the most notable feature is that they have an additional A’ strand (Fig. 1B–D). The two artiodactyl sCD8αα can be discriminated from each other by certain characteristics, such as the longer A’ strand and shorter complementarity determining region 2 (CDR2) loop in sCD8αα.

### Conserved interfacial aromatic residues are critical to CD8αα dimerization

**Figure 2:**
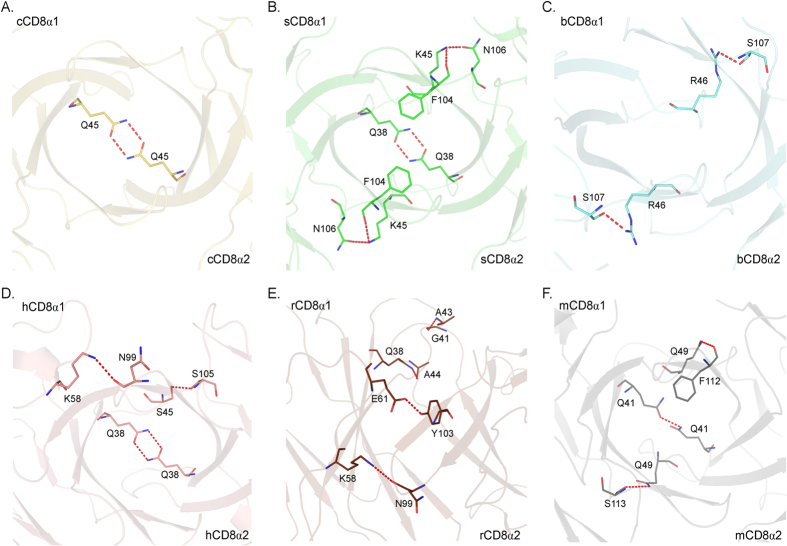
The inter-chain hydrogen bonds between the monomers of the CD8αα dimers. The CD8αα structures and colours are the same as above. The hydrogen bonds are shown as red dashed lines. (**A–F**) Hydrogen bonds in chicken, swine, bovine, human, monkey and mouse CD8αα homodimers. The residues forming these hydrogen bonds are shown in stick models. The numbers and locations of hydrogen bonds in the six CD8αα homodimers are not conserved.

The chicken cCD8αα structure further confirmed that the dimerization of CD8α is beyond that of mammals and was retained quite well during the evolution of endotherms. The buried surface areas (BSAs) of cCD8αα, sCD8αα, bCD8αα, mouse CD8αα (mCD8αα), rhesus macaque CD8αα (rCD8αα) and human CD8αα (hCD8αα) are 828.3 Å^2^, 979.6 Å^2^, 928.7 Å^2^, 1048.7 Å^2^, 1105.5 Å^2^ and 1026.3 Å^2^, respectively, indicating that they are tightly binding dimers. However, there are only a few hydrogen bonds formed by non-conserved residues between two monomers of these different species (Fig. 2). There are only 2~6 hydrogen bonds between the two monomers of these CD8αα dimers. The BSAs of sCD8αα and bCD8αα are similar, but there are six hydrogen bonds in sCD8αα and only two hydrogen bonds in bCD8αα, indicating that CD8α dimerization does not mainly rely on hydrogen bonds. The Q residue on the C strand is involved in the formation of hydrogen bonds in five CD8αα dimers, but it was substituted by H in bCD8α, and no hydrogen bond is formed at this location in bCD8αα (Figs 1D and 2).

**Figure 3:**
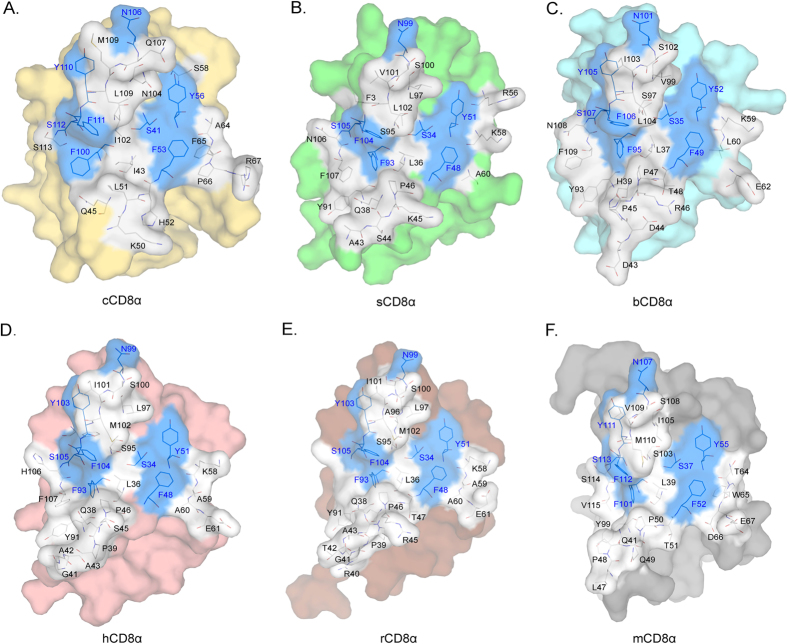
The residues in the interfaces of the six known CD8αα structures. Residues in the interfaces of the six known CD8αα homodimers are shown as stick models. The conserved residues are coloured blue, and non-conserved residues are coloured white. (**A–F**) The interfaces and residues composing chicken, swine, bovine, human, monkey and mouse CD8αα homodimers.

The residues on the dimer interface in the known CD8αα structures are shown in Fig. 3, and their contributions to dimerization are listed in Table S1. The numbers of residues in the interface of CD8αα dimers are approximately the same (26~27 residues). Among them, there are eight conserved residues, and their locations are invariable in the different CD8αα structures. Although the total van der Waals force (VDW) and BSA are variable among the different evolutionary CD8α, contributions by conserved residues are approximately the same. These interactions should be the basic insurance of CD8αα homodimerization. Among the conserved residues, the contributions of four aromatic AAs (F or Y) definitely account for absolute proportions. Three F in C’, as well as F and G strands and one Y in the C’ strand provide more than 100 VDW and 200 Å^2^ BSA. Especially for chicken, the four conserved aromatic residues contribute 161 VDW and 262.21 Å^2^ BSA, which account for 42% and 34% of the total VDW and BSA, respectively (Table S1). These four aromatic residues can interact with each other; for example, F48 contacts F104 in sCD8αα as well as in other CD8αα. Their inner interactions further ensure that dimerization can occur in a conserved manner during CD8α evolution. Therefore, the conserved aromatic AAs in the interface must be critical to preserving CD8αα homodimer formation during evolution.

### The binding of MHC I and CD8αα are anchored by three conserved residues in CD8α

**Figure 4:**
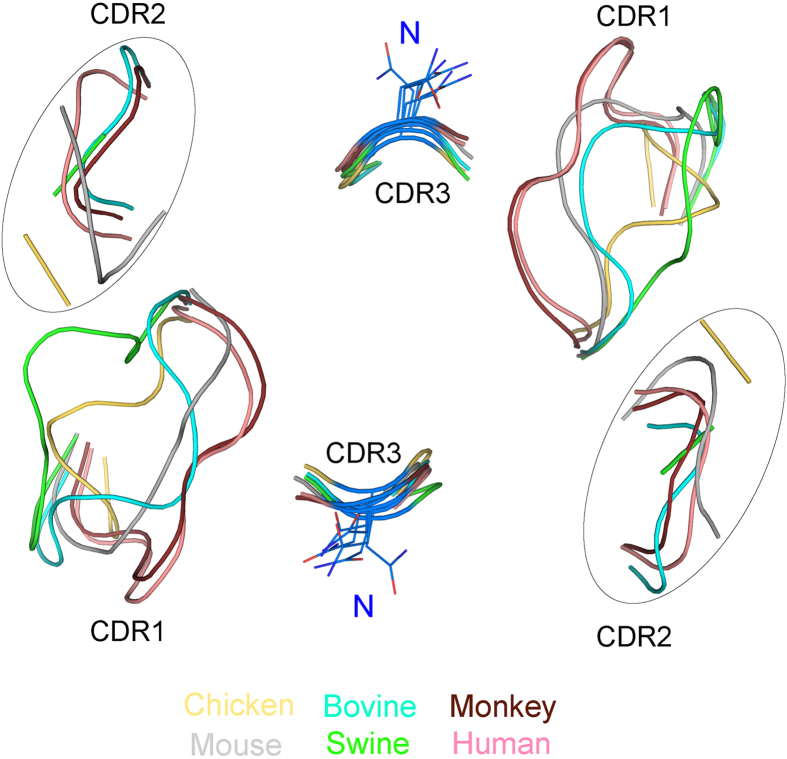
The diverse CDR1/2 and conserved CDR3 loops in CD8αα structures. The structural alignment shows the CDR-like loops of the six known CD8αα structures. Different colours consistent with those of the above figures were used to distinguish these six different CD8αα structures. The diverse CDR2-like loops were circled for clarity. The conserved residue N in the CDR3-like loop is shown in stick form.

It was generally believed that the CDR loops are the core functional domains in CD8α molecules because the human and mouse CD8αα-p/MHC I complex structures show that CD8αα uses three CDR loops to bind p/MHC I. The manner in which the two CD8αα-p/MHC I complexes bind is similar to that of antibody binding^25^,^26^,^27^. However, the structural alignment shows a great variation of the CDR1 and CDR2 loops in the known CD8αα structures (Fig. 4). The two loops showed diverse conformations and changed greatly in length, especially the CDR2 loops. The CDR2 loop of chicken cCD8αα only consists of two residues and has shifted dramatically compared with other CDR2 loops of mammalian CD8αα structures. In contrast, the CDR3 loops of the known structures showed great similarities in conformation and length, and there is only one conserved residue in the CDR3 loops (N in CDR3). This indicates that if the binding manner of CD8αα and p/MHC I is conserved during evolution, only the CDR3 loop, not CDR1 and CDR2, should be the principal part relating to binding MHC I molecules.

**Figure 5:**
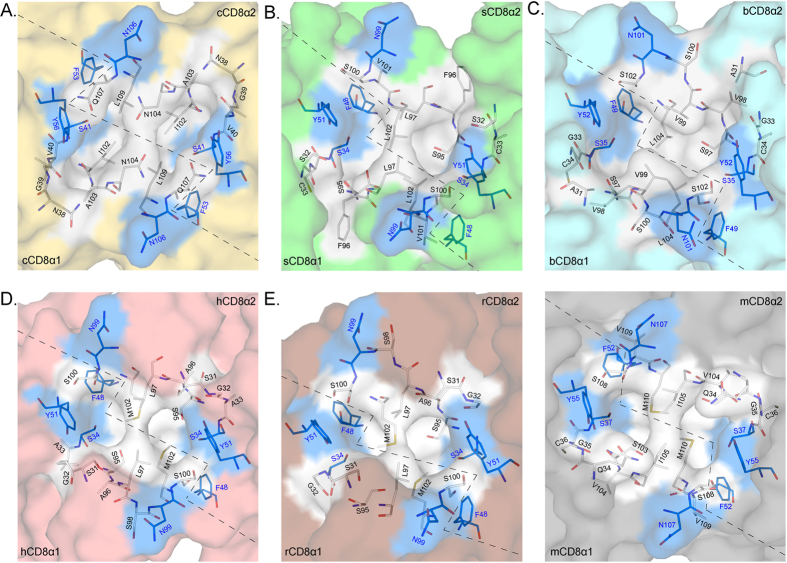
Compositions of binding cavities in the six known CD8αα structures. The cavities and residues of cCD8αα, sCD8αα, bCD8αα, hCD8αα, rCD8αα and mCD8αα homodimers are shown in (**A–F**) The conserved residues are coloured blue, and non-conserved residues are white. The two different monomers are separated by dashed line.

The cavity formed by CD8αα CDR loops accommodates an MHC I α3 domain CD loop, and it has been shown that they are the most important interacting parts^25^,^27^. The cavities of the six known CD8αα structures are shown in Fig. 5. The cavities are formed by identical residues separated symmetrically in the two monomers of each homodimer. The numbers of residues composing the cCD8αα, sCD8αα and bCD8αα cavities are 7, 7 and 9, and their volumes are 202.0 Å^3^, 322.9 Å^3^ and 384.6 Å^3^, respectively. Four conserved residues (S, F, Y from one monomer, and N from the other monomer) are involved in the cavities of CD8αα dimers; three of them (S, Y and N) are on the surface, and the other one (F) forms one side wall of the cavity. These conserved residues have been shown to interact MHC I by hydrogen bonds and are crucial for the binding.

**Figure 6:**
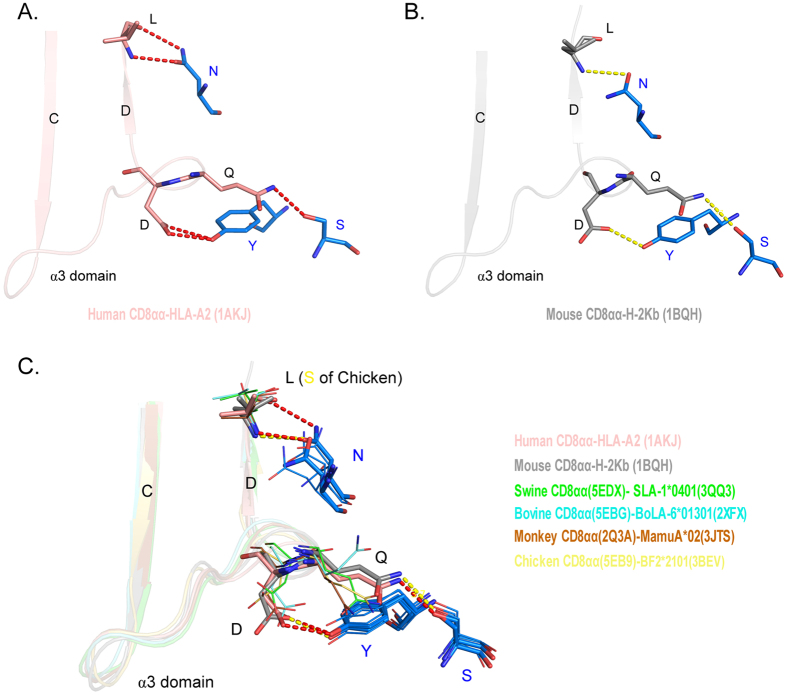
The key conserved residues maintain the interaction between CD8α and MHC I molecules. (**A**) The key conserved residues on the interface of human CD8αα and p/MHC I. The hydrogen bonds in the human MHC I-CD8αα complex (PDB ID: 1AKJ) are shown as red dashed lines. (**B**) The key conserved residues on the interface of mouse CD8αα and p/MHC I. The hydrogen bonds in the mouse MHC I-CD8αα complex (PDB ID: 1BQH) are shown as yellow dashed lines. (**C**) The conserved manner of interaction between p/MHC I and CD8αα. The MHC I and CD8αα of six different species are aligned according to human and mouse MHC I-CD8αα crystal structures. Human and mouse residues are shown in stick form, and chicken, swine, bovine and monkey residues are shown as lines. The CD8αα and p/MHC I structures used for the model are listed on the right side.

CD8 and MHC I are considered to be a pair of evolutionary molecules that maintain their relationship during co-evolution, and their co-evolutionary manner in endotherms was assessed using both the sequences and structures of six species (Fig. 6 and Figs S1 and S2). Regarding their sequences, the co-evolutionary relationships might not be obvious (Fig. S1). In human and mouse crystal structures of CD8αα-p/MHC I, three conserved residues of CD8α form hydrogen binds to MHC I α3 domain CD loop and D strand (Fig. 6A,B). Residues S and Y connect the side chains of D and Q in α3 CD loop with hydrogen bonds which are vital for the p/MHC I-CD8αα binding proved by mutation analysis. Residue N form hydrogen bonds with the main chain of L in D strand. The residues D and Q in α3 CD loop are highly conserved in MHC I molecular evolution. Residue L in α3 D strand is conserved in mammal MHC I molecules, although it changed into S in chicken MHC I, it can also form the hydrogen bonds with residues N in CD8α through its main chain atoms. By combination with chicken, swine, bovine and monkey MHC I and CD8αα, according to human and mouse CD8αα-p/MHC I binding way, we found their main chains are matched well and side chains are without steric hindrance (Fig. 6C). In addition, the structures of CD8αβ heterodimers of the rest five species were modelled based on the structure of mouse CD8αβ (Fig. S2). The interacting residues of CD8 and MHC I were further investigated, according to human and mouse CD8αα-p/MHC I and CD8αβ-p/MHC I crystal structures, and the interacting residues of MHC I were found to be more conserved than those of their partners, CD8αα and CD8αβ. Interestingly, only at the key interacting sites between CD8α and MHC I were the residues on their interface highly conserved (Fig. S2). In both CD8αα-p/MHC I and CD8αβ-p/MHC I complexes, these conserved residues on the CD8α surface form hydrogen bonds with the CD loop and D strand of the MHC I α3 domain. These results suggested that the binding manner of p/MHC I and CD8 was conserved and anchored by the residues conserved in them.

### Conserved residues in the outer and inner surfaces of CD8α molecules

**Figure 7:**
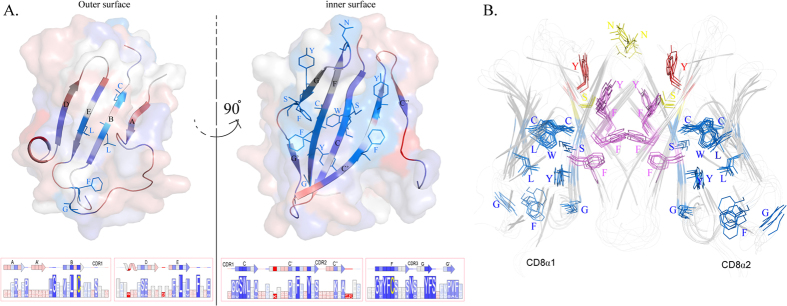
The distribution of conserved CD8α residues that are critical to guarantee its function. (**A**) cCD8α monomer was used as a model to show the distribution of conserved residues in CD8α. The outer surface contained A,B,E and D strands, and the inner surface contained the other strands of CD8α, which were coloured differently according to the conservation of the residues in them. The details of the residues in each position are shown in boxes with different sizes under the 3D surface illustration. (**B**) The classification of CD8α conserved residues based on their functions. All the conserved residues in the six CD8αα structures are shown in stick. The conserved residues which are critical to dimerization are coloured purple, and the residues taking part in the binding of MHC I according to elucidated human and mouse MHC I-CD8αα structures are coloured yellow. Only one conserved residue that plays a vital role in both dimerization and MHC I interaction is coloured red. The rest nine conserved CD8α residues not involved in these two aspects are coloured blue, and seven of them are common in other IgV molecules. The grouping indicates that only CD8α-specific conserved residues are critical to guarantee its continued function during evolution.

The conserved residues and their distribution in CD8α structures were coloured differently (using cCD8α as a model, Fig. 7A). Among all 17 conserved residues, only 4 residues are in loops, and the rest are in strands. From another perspective, the inner surface is more conserved than the outer surface of CD8α. There are only 5 conserved residues on the outer surface, and most of them (L and C in the B strand, L in the E strand and G in the AB loop) are common core components of IgV domains that are not special to CD8α^44^,^45^. Even in mammal CD8α molecules, the conservation of the outer surface was still less than that of the inner surface. However, there are 12 conserved residues in the inner surface, and 9 of them are special to CD8α. The conserved residues in the CDR3 loop are also located in the inner surface. The data suggested that conserved residues in the outer and inner surfaces are essential for the formation of CD8αα dimers and play critical roles in the core functions of CD8α conserved during evolution.

Because of the low AA identities of CD8α molecules from different species, there are only 17 conserved residues, and 7 of them are commonly conserved in IgV domains; only approximately 10 conserved residues were unique to CD8α. The results showed that the few uniquely conserved CD8α residues were critical to allow CD8α to form homodimers and interact with MHC I. Five conserved aromatic residues on the interface provide considerable intermolecular forces to ensure dimerization, and four conserved residues in the binding cavity form vital hydrogen bonds with p/MHC I. Residue Y in the C’ strand can play two different roles simultaneously, indicating it is essential for CD8α. The grouped of conserved residues is shown in Fig. 7B. The superposition of the conserved residues in all resolved CD8αα structures indicated that they are important for the maintenance of the proper form of CD8αα during evolution.

## Discussion

In this study, we first resolved the crystal structures of chicken, swine and bovine CD8αα and analysed all the known CD8αα structures to determine how CD8α could form a homodimer and bind p/MHC I.

The AA identities of CD8α molecules from different endotherm species were quite low (Fig. 1E); the identities between chicken and other mammalian CD8α sequences were even below 30%. So, all the resolved CD8αα structures showed their own significant specific characteristics. There was a special helix and a short CDR2 loop found in cCD8αα, and the inter-chain hydrogen bonds and the dimer interface area of cCD8αα were minimal. sCD8αα and bCD8αα showed an additional A’ strand (Fig. 1B–D), but the A’ strand in sCD8αα was longer. Even so, the CD8αα structures demonstrated that they are all homodimers formed by V-type immunoglobulins and have similar overall architectures. Additionally, we found that seven common conserved residues resulted in CD8α folding in a V-type conformation, and eight conserved residues of CD8 located on the dimer interface offered a large amount of VDW for the formation of homodimers. VDW and BSA provided by conserved residues in cCD8αα account were greater than 42% and 33% (Table S1), respectively, which were higher than those of sCD8αα and bCD8αα; these results indicated these conserved residues may be the initial key elements of CD8 dimerization. Four conserved aromatic residues offered the most VDW among all the conserved residues, indicating they play the most important role in homodimerization. In the structure of mouse CD8αβ (the only currently known heterodimer, PDB ID: 2ATP), there are 7 conserved residues on the interface of CD8α and six conserved residues of CD8β among these six species (Fig. S3). The four conserved aromatic residues we found in CD8α were also involved in the heterodimerization, and their BSA was 190 Å^2^, approximately 20% of the total amount of interface area. In CD8β, the total BSA of the conserved residues was 228 Å^2^, accounting for 25% of the total interacting area. These data suggested the conserved residues of CD8 (both CD8α and CD8β) are critical in dimerization^5^. Interestingly, there were also four conserved aromatic residues in CD8β, which provided the most BSA (221 Å^2^), and a sequence alignment showed three of them are identical to the key aromatic residues we found in CD8α. The only variation was in the chicken CD8β sequence, but the substituted residues had similar properties (Fig. S3).

The elucidated crystal structures of the human and mouse CD8αα-p/MHC I complexes suggested that the manners of CD8αα and MHC I interaction are very similar^25^,^27^. However, it was unclear that CD8αα from other species could bind p/MHC I in the same way. Based on the (modelled) structures from six species, we proposed that the interaction manner of CD8αα and p/MHC I is consistent and preserved by three completely conserved residues of CD8 during the evolution of endotherms. The compositions of CD8αα dimer binding cavities and three residues that play key roles in interacting with MHC I were highly conserved. These three residues are located on the surface of the cavities and can bind with MHC I strongly by hydrogen bonds and VDW. The residues in different MHC I molecules that are connected by the three residues were also highly conserved. Structural alignment showed that both of them are well superposed in the manner of CD8αα-p/MHC I interaction (Fig. 6). The three conserved residues acted as a three-point register, fixing the interaction of CD8αα and p/MHC I in a consistent manner during evolution. In the crystal structures of the CD8αα-TL (PDB ID: 1NEZ) and CD8αβ-p/MHC I complexes, these three residues could also form the same hydrogen bonds as in the CD8αα-MHC I complex^16^. Moreover, in CD8αβ-p/MHC I (PDB ID: 3DMM), CD8β occupies a T-cell membrane proximal position and mainly interacts with the CD loop of the MHC I α3 domain. However, the conservation of residues in this region was not as high as in CD8α (Fig. S2). The structures showed that CD8α is at the same position and binds MHC I in the same way in both the CD8αα-p/MHC I and CD8αβ-p/MHC I complexes. These results strongly indicated that both CD8 isoforms maintain the manner of binding MHC I by relying on the conserved residues of CD8α.

CD8α and p/MHC I are considered as a set of co-evolution molecules because their binding is critical to CTL immunity in vertebrate species. The co-evolution relationship is not obvious by their AA sequences alone, and the interacting residues of MHC I were found to be more conserved than those of their CD8 partners (Fig. S2). However, the key residues for complex binding are highly conserved in both molecules according to the elucidated and modelled MHC I-CD8αα structures (Fig. 6). In consideration of the weak binding affinity between CD8αα and MHC I^6^, we posit that a few conserved residues playing critical roles in this interaction are enough to ensure the binding continues during evolution.

In this study, crystal structures of CD8αα and p/MHC I from six different species were analysed. The structures and sequences were significantly different between these six species, especially between chickens and mammals; however, they indicate that a few key conserved residues could ensure the structural basis of CD8αα dimerization and binding with p/MHC I via great sequence variations during endotherm evolution.

## Materials and Methods

### Preparation of proteins

The genes encoding cCD8α, sCD8α and bCD8α mature peptides (extracellular IgV domains) were chemically synthesized and ligated into a pET21a vector (Novagen) by the Shanghai Generay Biotechnology Company according to the sequences in GeneBank (NM_205235, NM_001001907 and NM_174015). The plasmids were transformed into the *Escherichia coli* strain BL21 (DE3), and 0.5 mM IPTG was used to induce the expression of these three inclusion bodies^46^. The bacteria were harvested by centrifugation at 6 000 g for 10 min and were then resuspended in cold phosphate-buffered saline (PBS). After sonication, the samples were centrifuged at 16 000 g, and the pellets were washed three times with a solution consisting of 20 mM Tris–HCl pH 8.0, 100 mM NaCl, 1 mM EDTA, 1 mM DTT and 0.5% Triton X-100. Finally, the inclusion bodies were dissolved in guanidinium chloride (Gua–HCl) buffer [6 M Gua–HCl, 50 mM Tris–HCl pH 8.0, 10 mM EDTA, 100 mM NaCl, 10% (v/v) glycerine, 10 mM DTT] to a concentration of 30 mg ml^−1^.

### Refolding and purification

The dissolved cCD8α, sCD8α and bCD8α inclusion bodies were gradually added into refolding buffer (100 mM Tris-HCl, 2 mM EDTA, 400 mM L-arginine-HCl, 0.5 mM oxidised glutathione, 5 mM reduced glutathione, pH 7.4) until a concentration of 60 mg ml^−1^ was reached. After incubation for 24 h at 277 K, the soluble portions were concentrated and purified by chromatography on a Superdex 75 10/300 column (GE Healthcare). The eluted peaks were collected by 0.5 ml per tube and tested by SDS-PAGE. Then, the refolded cCD8α, sCD8α and bCD8α were pooled together.

### Crystallisation

Crystals of sCD8α were obtained as described previously^42^. The purified cCD8α and bCD8α were concentrated to 5 mg ml^−1^ and 10 mg ml^−1^ in a buffer containing 20 mM Tris (pH 7.4) and 50 mM NaCl for crystallisation. After being mixed with reservoir buffer at a 1:1 ratio, cCD8α and bCD8α were crystallised in solution using PEGIon^TM^ kit (Hampton Research, Riverside, CA) No. 23 (0.2 M Ammonium formate, 20% w/v Polyethylene glycol 3,350) and Index^TM^ kit (Hampton Research, Riverside, CA) No. 67 (0.2 M Ammonium sulphate, 0.1 M Bis-Tris pH 6.5, 20% w/v Polyethylene glycol 3,350), respectively by the hanging-drop vapour diffusion technique at 291 K.

### Data collection and processing

Diffraction data of three different CD8α crystals were collected using the NE3A beamline at the KEK synchrotron facility (Tsukuba, Japan) and an ADSC Q270 imaging-plate detector at a wavelength of 1.0 Angstrom, the BSRF 3W1A beamline and an MAR scanner 345-mm plate at a wavelength of 1.0 Angstrom, and a Rigaku MicroMax-007 HF and Rigaku Raxis IV++ at a wavelength of 1.54178 Angstrom, respectively. In each case, the crystals were first soaked in reservoir solution containing 15% glycerol as a cryoprotectant for several seconds and then flash-cooled in a stream of gaseous nitrogen at 100 K^47^. The collected intensities were indexed, integrated, corrected for absorption, scaled and merged using HKL2000^48^.

### Structure determination and refinement

The structures of cCD8α, sCD8α and bCD8α were resolved by molecular replacement using the MOLREP programme with human CD8α (PDB code: 1CD8) as the search model. Extensive model building was performed by hand using COOT^49^, and restrained refinement was performed using REFMAC5. Further rounds of refinement were performed using the phenix.refine programme implemented in the PHENIX package with isotropic ADP refinement and bulk solvent modelling^50^. The stereochemical quality of the final model was assessed with the PROCHECK programme^51^. Data collection and refinement statistics are listed in Table 1.

### PDB accession numbers

The crystal structures have been deposited in the Protein Data Bank (http://www.pdb.org/pdb/home/home.do) with accession 5EB9 (cCD8α), 5EDX (sCD8α) and 5EBG (bCD8α), respectively.

### Sequence alignment and structural analysis

The alignment of CD8α AAs sequences was completed by the ClustalW2 server (http://www.ebi.ac.uk/Tools/msa/clustalw2/). The structural analyses were completed by the PDBePISA (http://www.ebi.ac.uk/msd-srv/prot_int/pistart.html) and CASTp servers (http://sts-fw.bioengr.uic.edu/castp/about.php) and the PyMOL (DeLano Scientific LLC) and CCP4 programmes.

## Additional Information

**How to cite this article**: Liu, Y. *et al*. The structural basis of chicken, swine and bovine CD8αα dimers provides insight into the co-evolution with MHC I in endotherm species. *Sci. Rep*. **6**, 24788; doi: 10.1038/srep24788 (2016).

## Supplementary Material

Supplementary Information

## Figures and Tables

**Table 1:** X-ray diffraction data processing and refinement statistics.

	cCD8αα	sCD8αα	bCD8αα
Data processing			
Space group	C2221	P3221	P6_1_22
Cell parameters (Å)	a = 45.36, b = 87.06, c = 70.31	a = 80.97, b = 80.97, c = 95.19	a = 74.42, b = 74.42, c = 143.29
Resolution range (Å)	50.00–2.00	50.00–1.80	50.00–1.80
Total reflections	47439	516532	464682
Unique reflections	9124	33859	21811
Completeness (%)	97.5 (78.5)^a^	99.9 (99.9)^a^	99.9 (99.9)^a^
R_merge_ (%)^b^	7.3 (33.6)	10.7 (58.9)^a^	8.6 (48.4)^a^
I/σ	20.62 (2.824)	30.24 (5.48)^a^	41.00 (5.36)^a^
Refinement			
R factor (%)^c^	23.8	18.4	19.9
R_free_ (%)	26.3	20.9	21.7
r.m.s. deviation			
Bonds (Å)	0.016	0.008	0.010
Angles (°)	1.364	0.997	1.159
Average B factor	37.736	23.769	17.321
Most favored (%)	87.6	88.3	89.8
Disallowed (%)	0.0	0.0	0.0

^a^Numbers in parentheses correspond to the highest resolution shell. r.m.s.d., Root-mean-square deviations from ideal geometry.

^b^Rmerge = ∑h∑Iih − <Ih>/∑h∑I<Ih>, where <Ih> is the mean intensity of the observations Iih of reflection h.

^c^R factor = ∑(Fobs − Fcalc)/∑Fobs; Rfree is the R factor for a subset (5%) of reflections that was selected prior to refinement calculations and not included in the refinement.

## References

[b1] Cooper M. D. & Alder M. N. The evolution of adaptive immune systems. Cell 124, 815–822 (2006). 16497590 10.1016/j.cell.2006.02.001

[b2] Litman G. W., Cannon J. P. & Dishaw L. J. Reconstructing immune phylogeny: new perspectives. Nature reviews. Immunology 5, 866–879 (2005). 10.1038/nri1712 PMC3683834 16261174

[b3] Venkatesh B. . Elephant shark genome provides unique insights into gnathostome evolution. Nature 505, 174–179 (2014). 24402279 10.1038/nature12826 PMC3964593

[b4] Nakanishi T., Shibasaki Y. & Matsuura Y. T. Cells in Fish. Biology (Basel) 4, 640–663 (2015). 26426066 10.3390/biology4040640 PMC4690012

[b5] Chang H. C. . Structural and mutational analyses of a CD8alphabeta heterodimer and comparison with the CD8alphaalpha homodimer. Immunity 23, 661–671 (2005). 16356863 10.1016/j.immuni.2005.11.002

[b6] Gao G. F. & Jakobsen B. K. Molecular interactions of coreceptor CD8 and MHC class I: the molecular basis for functional coordination with the T-cell receptor. Immunology today 21, 630–636 (2000). 11114424 10.1016/s0167-5699(00)01750-3

[b7] Kieffer L. J. . Identification of a candidate regulatory region in the human CD8 gene complex by colocalization of DNase I hypersensitive sites and matrix attachment regions which bind SATB1 and GATA-3. Journal of immunology 168, 3915–3922 (2002). 10.4049/jimmunol.168.8.3915 11937547

[b8] Wooldridge L. . MHC class I molecules with Superenhanced CD8 binding properties bypass the requirement for cognate TCR recognition and nonspecifically activate CTLs. Journal of immunology 184, 3357–3366 (2010). 10.4049/jimmunol.0902398 PMC3024536 20190139

[b9] Holler P. D. & Kranz D. M. Quantitative analysis of the contribution of TCR/pepMHC affinity and CD8 to T cell activation. Immunity 18, 255–264 (2003). 12594952 10.1016/s1074-7613(03)00019-0

[b10] Janeway C. A. Jr. The T cell receptor as a multicomponent signalling machine: CD4/CD8 coreceptors and CD45 in T cell activation. Annual review of immunology 10, 645–674 (1992). 10.1146/annurev.iy.10.040192.003241 1534242

[b11] Veillette A., Bookman M. A., Horak E. M. & Bolen J. B. The CD4 and CD8 T cell surface antigens are associated with the internal membrane tyrosine-protein kinase p56lck. Cell 55, 301–308 (1988). 3262426 10.1016/0092-8674(88)90053-0

[b12] Rybakin V., Clamme J. P., Ampudia J., Yachi P. P. & Gascoigne N. R. CD8alphaalpha and -alphabeta isotypes are equally recruited to the immunological synapse through their ability to bind to MHC class I. EMBO reports 12, 1251–1256 (2011). 22081144 10.1038/embor.2011.209 PMC3245696

[b13] Gangadharan D. & Cheroutre H. The CD8 isoform CD8alphaalpha is not a functional homologue of the TCR co-receptor CD8alphabeta. Current opinion in immunology 16, 264–270 (2004). 15134773 10.1016/j.coi.2004.03.015

[b14] Wong J. S. . Stalk region of beta-chain enhances the coreceptor function of CD8. Journal of immunology 171, 867–874 (2003). 10.4049/jimmunol.171.2.867 12847256

[b15] Moody A. M. . Developmentally regulated glycosylation of the CD8alphabeta coreceptor stalk modulates ligand binding. Cell 107, 501–512 (2001). 11719190 10.1016/s0092-8674(01)00577-3

[b16] Arcaro A. . CD8beta endows CD8 with efficient coreceptor function by coupling T cell receptor/CD3 to raft-associated CD8/p56(lck) complexes. J Exp Med 194, 1485–1495 (2001). 11714755 10.1084/jem.194.10.1485 PMC2193676

[b17] Wang R., Natarajan K. & Margulies D. H. Structural basis of the CD8 alpha beta/MHC class I interaction: focused recognition orients CD8 beta to a T cell proximal position. Journal of immunology 183, 2554–2564 (2009). 10.4049/jimmunol.0901276 PMC2782705 19625641

[b18] Cheroutre H. & Lambolez F. Doubting the TCR coreceptor function of CD8alphaalpha. Immunity 28, 149–159 (2008). 18275828 10.1016/j.immuni.2008.01.005

[b19] Olivares-Villagomez D. & Van Kaer L. TL and CD8alphaalpha: Enigmatic partners in mucosal immunity. Immunology letters 134, 1–6 (2010). 20850477 10.1016/j.imlet.2010.09.004 PMC2967663

[b20] Liu Y. . The crystal structure of a TL/CD8alphaalpha complex at 2.1 A resolution: implications for modulation of T cell activation and memory. Immunity 18, 205–215 (2003). 12594948 10.1016/s1074-7613(03)00027-x

[b21] Masopust D., Vezys V., Wherry E. J., Barber D. L. & Ahmed R. Cutting edge: gut microenvironment promotes differentiation of a unique memory CD8 T cell population. Journal of immunology 176, 2079–2083 (2006). 10.4049/jimmunol.176.4.2079 16455963

[b22] Madakamutil L. T. . CD8alphaalpha-mediated survival and differentiation of CD8 memory T cell precursors. Science 304, 590–593 (2004). 15105501 10.1126/science.1092316

[b23] Leahy D. J., Axel R. & Hendrickson W. A. Crystal structure of a soluble form of the human T cell coreceptor CD8 at 2.6 A resolution. Cell 68, 1145–1162 (1992). 1547508 10.1016/0092-8674(92)90085-q

[b24] Zong L. . Rhesus macaque: a tight homodimeric CD8alphaalpha. Proteins 75, 241–244 (2009). 19137599 10.1002/prot.22331

[b25] Kern P. S. . Structural basis of CD8 coreceptor function revealed by crystallographic analysis of a murine CD8alphaalpha ectodomain fragment in complex with H-2Kb. Immunity 9, 519–530 (1998). 9806638 10.1016/s1074-7613(00)80635-4

[b26] Shi Y., Qi J., Iwamoto A. & Gao G. F. Plasticity of human CD8alphaalpha binding to peptide-HLA-A*2402. Molecular immunology 48, 2198–2202 (2011). 21645925 10.1016/j.molimm.2011.05.009

[b27] Gao G. F. . Crystal structure of the complex between human CD8alpha(alpha) and HLA-A2. Nature 387, 630–634 (1997). 9177355 10.1038/42523

[b28] Liaw H. J. . Genomic organization of the chicken CD8 locus reveals a novel family of immunoreceptor genes. Journal of immunology 178, 3023–3030 (2007). 10.4049/jimmunol.178.5.3023 17312148

[b29] Chakraborty A. K. & Das S. K. Molecular cloning and characterization of the guinea pig cholinephosphotransferase gene. Biochemical and biophysical research communications 312, 1104–1110 (2003). 14651986 10.1016/j.bbrc.2003.11.033

[b30] Luhtala M., Tregaskes C. A., Young J. R. & Vainio O. Polymorphism of chicken CD8-alpha, but not CD8-beta. Immunogenetics 46, 396–401 (1997). 9271629 10.1007/s002510050293

[b31] Lalor P. . Molecular cloning, reconstruction and expression of the gene encoding the alpha-chain of the bovine CD8–definition of three peptide regions conserved across species. Immunology 76, 95–102 (1992). 1628904 PMC1421739

[b32] Gerner W., Kaser T. & Saalmuller A. Porcine T lymphocytes and NK cells–an update. Developmental and comparative immunology 33, 310–320 (2009). 18601948 10.1016/j.dci.2008.06.003

[b33] Yang H. & Parkhouse R. M. Differential expression of CD8 epitopes amongst porcine CD8-positive functional lymphocyte subsets. Immunology 92, 45–52 (1997). 9370923 10.1046/j.1365-2567.1997.00308.x PMC1363980

[b34] Taylor B. C., Dellinger J. D., Cullor J. S. & Stott J. L. Bovine milk lymphocytes display the phenotype of memory T cells and are predominantly CD8+. Cellular immunology 156, 245–253 (1994). 8200039 10.1006/cimm.1994.1169

[b35] Zhang J. . Narrow groove and restricted anchors of MHC class I molecule BF2*0401 plus peptide transporter restriction can explain disease susceptibility of B4 chickens. Journal of immunology 189, 4478–4487 (2012). 10.4049/jimmunol.1200885 PMC5018395 23041567

[b36] Zhang N. . Crystal structure of swine major histocompatibility complex class I SLA-1 0401 and identification of 2009 pandemic swine-origin influenza A H1N1 virus cytotoxic T lymphocyte epitope peptides. Journal of virology 85, 11709–11724 (2011). 21900158 10.1128/JVI.05040-11 PMC3209268

[b37] Li X. . Two distinct conformations of a rinderpest virus epitope presented by bovine major histocompatibility complex class I N*01801: a host strategy to present featured peptides. Journal of virology 85, 6038–6048 (2011). 21450819 10.1128/JVI.00030-11 PMC3126294

[b38] Macdonald I. K. . MHC class I bound to an immunodominant Theileria parva epitope demonstrates unconventional presentation to T cell receptors. Plos pathogens 6, 404–408 (2010). 10.1371/journal.ppat.1001149 PMC2954893 20976198

[b39] Koch M. . Structures of an MHC class I molecule from B21 chickens illustrate promiscuous peptide binding. Immunity 27, 885–899 (2007). 18083574 10.1016/j.immuni.2007.11.007

[b40] Wang Z. . Complex assembly, crystallization and preliminary X-ray crystallographic analysis of the bovine CD8alphaalpha-BoLA-2*02201 complex. Acta Crystallogr F Struct Biol. Commun 70, 742–746 (2014). 24915083 10.1107/S2053230X14008838 PMC4051527

[b41] Liu Y., Chen R., Tariq M. & Xia C. Complex assembly, crystallization and preliminary X-ray crystallographic analysis of the chicken CD8alphaalpha-BF2*0401 complex. Acta Crystallogr F Struct Biol Commun 70, 1264–1267 (2014). 25195906 10.1107/S2053230X14017154 PMC4157433

[b42] Zhang N. . Crystallization and preliminary X-ray crystallographic studies of swine CD8alpha. *Acta crystallographica*. Section F, Structural biology and crystallization communications 67, 888–891 (2011). 10.1107/S1744309111020392 PMC3151120 21821887

[b43] Flajnik M. F. & Kasahara M. Comparative genomics of the MHC: glimpses into the evolution of the adaptive immune system. Immunity 15, 351–362 (2001). 11567626 10.1016/s1074-7613(01)00198-4

[b44] Halaby D. M., Poupon A. & Mornon J. The immunoglobulin fold family: sequence analysis and 3D structure comparisons. Protein engineering 12, 563–571 (1999). 10436082 10.1093/protein/12.7.563

[b45] Bork P., Holm L. & Sander C. The immunoglobulin fold. Structural classification, sequence patterns and common core. Journal of molecular biology 242, 309–320 (1994). 7932691 10.1006/jmbi.1994.1582

[b46] Cole D. K. . Crystallization and preliminary X-ray structural studies of a high-affinity CD8alphaalpha co-receptor to pMHC. *Acta crystallographica*. Section F, Structural biology and crystallization communications 61, 285–287 (2005). 10.1107/S1744309105002988 PMC1952291 16511019

[b47] Parkin S. & Hope H. Macromolecular cryocrystallography: cooling, mounting, storage and transportation of crystals. J. Appl. Crystallogr 31, 945–953 (1998).

[b48] Otwinowski & Minor. Processing of X-ray Diffraction Data Collected in Oscillation Mode. Methods Enzymol 276, 307–326 (1997). 10.1016/S0076-6879(97)76066-X 27754618

[b49] Emsley P. & Cowtan K. Coot: model-building tools for molecular graphics. Acta crystallographica. Section D, Biological crystallography 60, 2126–2132 (2004). 15572765 10.1107/S0907444904019158

[b50] Adams P. D. . PHENIX: building new software for automated crystallographic structure determination. Acta crystallographica. Section D, Biological crystallography 58, 1948–1954 (2002). 12393927 10.1107/s0907444902016657

[b51] Laskowski R. A., Moss D. S. & Thornton J. M. Main-chain bond lengths and bond angles in protein structures. Journal of molecular biology 231, 1049–1067 (1993). 8515464 10.1006/jmbi.1993.1351

