# Supplementary material for: The structural basis of chicken, swine and bovine CD8αα dimers provides insight into the co-evolution with MHC I in endotherm species
Source: Sci Rep. 2016 Apr 28;6:24788. doi: 10.1038/srep24788 (PMC4848529; doi:10.1038/srep24788)
Supplement: Supplementary Information [file srep24788-s1.pdf]

The structural basis of chicken, swine and bovine CD8αα dimers provides insight into the co-evolution with MHC I in endotherm species

Yanjie Liu[1,3], Xin Li[1], Jianxun Qi[4], Nianzhi Zhang[1]*, and Chun Xia[1,2]*

Corresponding author: Prof. Chun Xia or Nianzhi Zhang

E-mail: xiachun@cau.edu.cn

Address: Department of Microbiology and Immunology, College of Veterinary Medicine,

China Agricultural University, Beijing 100094, China

Table S1. Contributions of CD8αα dimerization by residues in interface

	Chicken			Bovine			Swine		
	Residue	VDW*	BSA (Å^2)*	Residue	VDW	BSA (Å^2)*	Residue	VDW	BSA (Å^2)*
conserved	S41	5	13.74	S35	3	7.74	S34	4	11.84
	F53	55	66.95	F49	58	74.02	F48	58	64.10
	Y56	28	41.16	Y52	24	37.60	Y51	28	28.91
	F100	16	35.34	F95	25	28.09	F93	6	9.54
	N106	1	3.44	N101	2	17.67	N99	5, H*	26.66
	Y110	11	29.09	Y105	0	1.14	Y103	0	0
	F111	51	89.67	F106	37	76.54	F104	54, H	98.23
	S112	0	1.84	S107	12, H	9.62	S105	15	6.64
Total		167	281.23		161	252.42		170	245.92
Non-conserved	F34	0	1.10	H39	15	26.47	F3	0	0.15
	L43	10	18.92	D43	4	7.90	L36	11	21.92
	Q45	13	35.11	D44	16	11.25	Q38	13, H	25.91
	G49	4, H*	9.68	P45	49	102.71	A43	21	50.71
	K50	0	1.67	R46	74, H	109.04	S44	23	13.00
	L51	38	93.64	P47	33	61.65	K45	H	96.39
	H52	7	1.12	T48	3	0.17	P46	43	64.83
	S58	0	0.17	K59	9	43.03	R56	6	15.77
	A64	5	28.19	L60	2	3.19	K58	18	48.39
	F65	6	16.07	A61	1	3.52	A60	0	1.67
	Y66	5	7.53	E62	0	2.61	Y91	8	21.42
	R67	21	52.77	Y93	16	16.53	S95	2	7.03
	I102	3	22.71	S97	2	4.19	L97	1	32.22
	N104	5	21.17	V99	0	4.86	S100	48	90.63
	Q107	37	74.49	S102	29	63.46	V101	29	52.06
	M108	37	81.42	I103	30	69.50	L102	38	94.99
	L109	36	81.18	L104	39	93.93	N106	22, H	28.01
	S113	0	0.17	N108	18	13.53	F107	58	63.03
				F109	56	65.14			
Total		227	547.11		402	702.68		410	728.13

	Human			Monkey			Mouse		
	Residue	VDW*	BSA (Å^2)*	Residue	VDW	BSA (Å^2)*	Residue	VDW	BSA (Å^2)*
conserved	S34	9	13.45	S34	3	14.15	S37	5	12.80
	F48	74	65.73	F48	69	77.34	F52	58	61.22
	Y51	28	27.72	Y51	21	29.28	Y55	22	30.17
	F93	10	9.54	F93	8	7.19	F101	8	12.07
	N99	18	16.57	N99	6, H*	16.14	N107	3	4.05
	Y103	5	6.25	Y103	6, H*	18.49	Y111	0	2.62
	F104	60	75.58	F104	50	83.42	F112	37	71.50
	S105	10	10.01	S105	7	8.89	S113	6, H*	11.16
Total		214	224.85		170	254.90		139	205.59
Non-conserved	S1	1	0	N1	1	4.55	L39	13	30.21
	L36	12	19.41	L36	7	20.50	Q41	23, H	34.06
	Q38	51, H	20.71	Q38	27	30.20	L47	21	66.20
	R40	34	32.46	G41	26, H	38.68	P48	49	93.63
	G41	45	36.32	T42	32, H	58.62	Q49	36, H	63.95
	A42	52	59.97	A43	33	52.05	P50	38	60.33
	A43	14	15.98	A44	21, H	15.40	T51	4	0.84
	A44	18	2.28	R45	45	89.26	K62	1	10.16
	S45	67	68.03	P46	31	73.97	T64	8	30.86
	P46	47	60.80	T47	7	1.46	W65	0	1.84
	T47	5	0	K58	20	42.33	D66	0	6.56
	K58	38	51.92	A59	7	2.33	E67	11	40.25
	A59	2	0.24	A60	4	6.03	Y99	27	43.00
	A60	1	5.86	Q61	2	22.44	S103	4	3.70
	E61	15	22.50	Y91	12	22.18	I105	2	35.82
	Y91	18	16.62	S95	3	6.54	S108	45, H	76.76
	S95	5	9.96	L97	1	19.99	V109	26	51.20
	A96	0	1.93	S100	34	69.14	M110	47	100.74
	L97	1	31.54	I101	39	88.36	S114	11	10.80
	S100	44	65.48	M102	45	94.79	V115	35, H	59.85
	I101	52	82.73	H106	28	36.72	P117	5	22.33
	M102	58	116.5	F107	54	55.01			
	H106	15	9.11						
	F107	89	71.09						
Total		684	801.44		479	850.55		407	843.09

*VDW: Van der Waal force

*H: hydrogen bond

*BSA: buried surface area

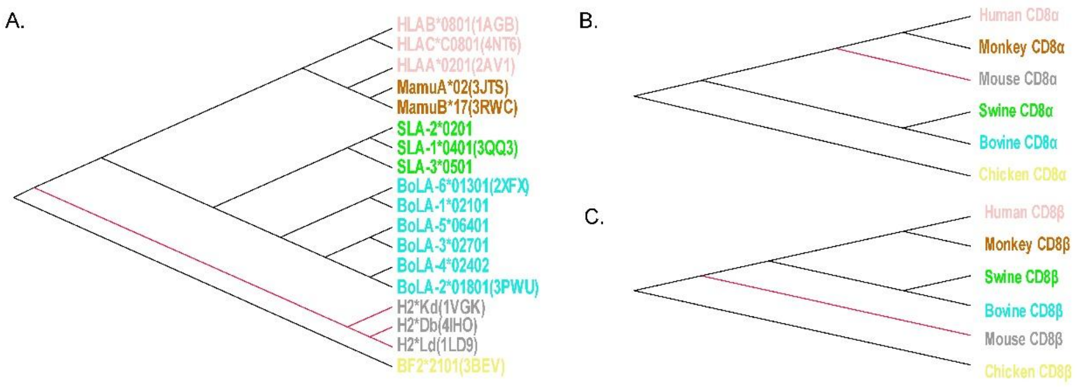

Fig. S1. The phylogenetic trees of CD8 and MHC I molecules of six endotherm species with elucidated crystal structures.

A. The phylogenetic trees of MHC I molecules from different six species. The MHC I alleles with known crystal structure were labelled by their PDB ID. **B.** The phylogenetic trees of CD8α molecules. **C.** The phylogenetic trees of CD8β molecules.

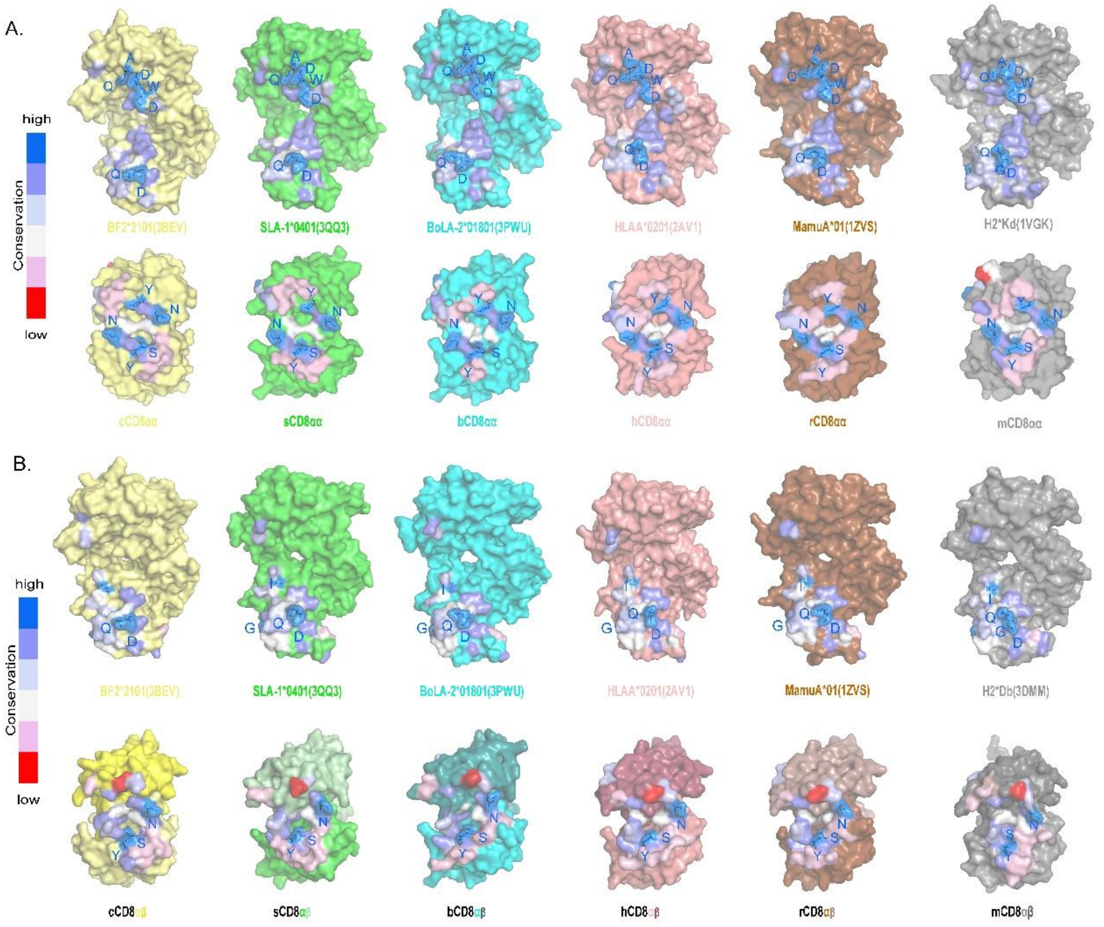

Fig. S2. Residues and the conservation in the interfaces of chicken, swine, bovine, macaque, monkey and human CD8αα/CD8αβ and p/MHC I

The completely conserved residues are labelled and shown in stick form. The interface between the CD8 dimers and MHC I are coloured differently according to the conservations of these residues in the six known CD8α structures. **A.** Interface of CD8αα and MHC I. **B.** Interface of CD8αβ and MHC I.

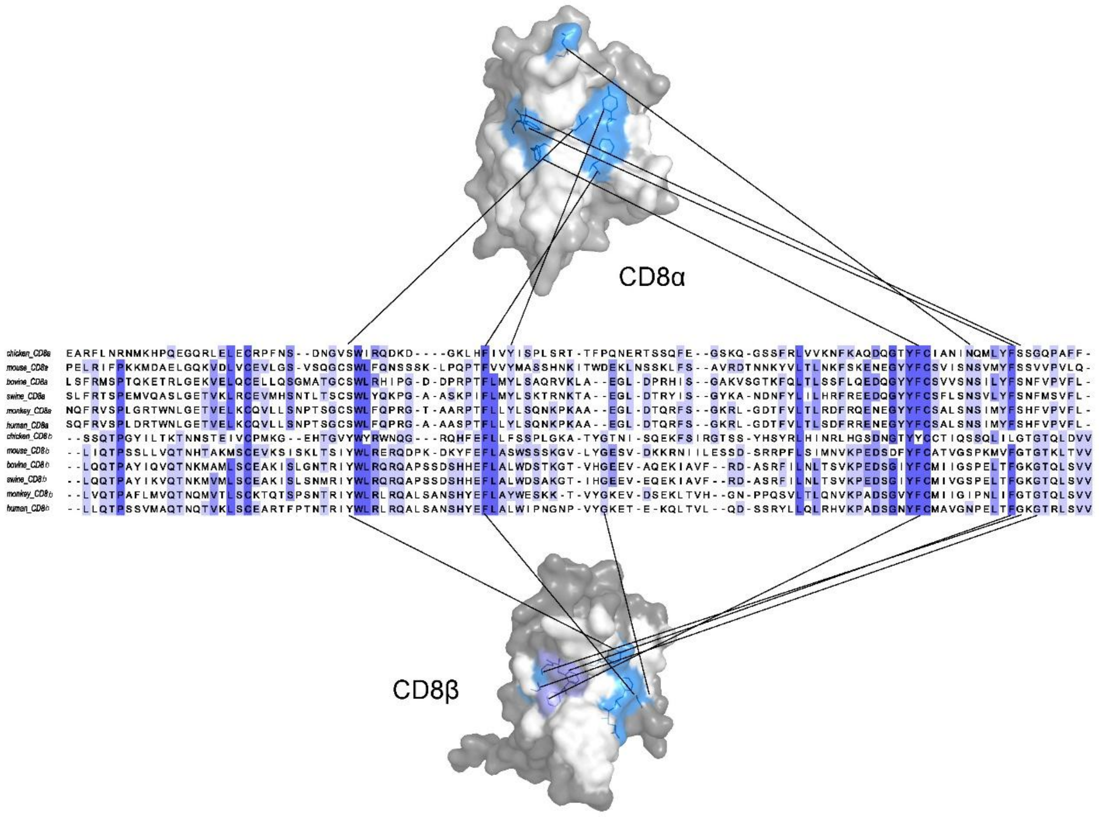

Fig. S3. The conservation of residues on the CD8αβ interface

Sequences of CD8α and CD8β are aligned to show their conservation. Residues on the interface of mouse CD8αβ are labelled with different colours to indicate their conservations. Residues in the structures were linked to their sequence positions by lines.
